# Integration of the IDDSI Scale into 3D Food Printing: A Strategy to Improve Food Safety and Quality of Life for People with Dysphagia

**DOI:** 10.3390/nu17243925

**Published:** 2025-12-15

**Authors:** Daniel García-Gutiérrez, Bartomeu Ayala Márquez, Xavier Gironés García, Ana Molero Muñoz, Cristina García-Salido, Estel·la Ramírez-Baraldes, Yirsa Jiménez-Pérez

**Affiliations:** 1 Department of Nursing, Faculty of Health Sciences at Manresa, Universitat de Vic-Universitat Central de Catalunya (UVic-UCC), Av. Universitària 4-6, 08242 Manresa, Barcelona, Spain; 2 Research Group in Investigación en Simulación e Innovación Transformativa (GRIST), Instituto de Investigación e Innovación en Ciencias de la Vida y de la Salud de la Cataluña Central (Iris-CC), Ctra. De Roda Núm. 70, 08500 Vic, Barcelona, Spain; 3 Department of Health and Social Innovation, Althaia Xarxa Assistencial Universitària de Manresa, Fundación Privada, Carrer Dr. Joan Soler, 1-3, 08242 Manresa, Barcelona, Spain; 4 Department of Research, Consorcio Sanitari de Terrassa, Carretera de Torrebonica, s/n, 08227 Terrassa, Barcelona, Spain; 5 Department of Rapid Diagnosis in Otolaryngology, Consorci Sanitari Integral, Hospital de Sant Joan Despí, Complex Hospitalari Universitari Moisès Broggi, Carrer d’Oriol Martorell, 12, 08970 Sant Joan Despí, Barcelona, Spain; 6 Intensive Care Unit, Althaia Xarxa Assistencial Universitària de Manresa, Fundació Privada, Carrer Dr. Joan Soler, 1-3, 08242 Manresa, Barcelona, Spain; 7 Department of Social Psychology and Quantitative Psychology, Universitat de Barcelona (UB), Passeig Vall d’Hebron, 171 Campus Mundet, 08035 Barcelona, Spain; 8 Department of Psychology, Universitat de Vic-Universitat Central de Catalunya (UVic-UCC), Carrerde la Laura, 13, 08500 Vic, Barcelona, Spain

**Keywords:** IDDSI Scale, validation, swallowing disorders, 3D printing, nutrition, quality of life, nursing care

## Abstract

Background: Dysphagia negatively impacts quality of life and requires diets with specifically modified textures. The IDDSI (International Dysphagia Diet Standardization Initiative) scale provides standardized criteria to ensure food safety. This research aims to explore and validate the IDDSI scale adapted to the consumption of foods developed with 3D printing in patients with dysphagia. Methodology: Different dishes were designed and validated using 3D printing and were evaluated by both healthcare professionals and people with dysphagia. In the second phase, participants analyzed their texture using the IDDSI scale. A mixed methodological approach was applied, combining quantitative data (from validated scales) and qualitative data (obtained through interviews and focus groups), ensuring methodological triangulation. Methods: In the first phase of the study, different dishes were cooked and designed using 3D printing technology and were previously evaluated by both healthcare professionals and people with dysphagia. In the second phase, all the dishes validated in the first phase were analyzed and classified according to their texture using the IDDSI. Results: A total of 24 dishes, backed by 204 validations, were determined to be suitable for people with dysphagia and compatible with 3D printing. According to the IDDSI analysis, 36% of these dishes were classified as level 3 (soft texture) and 64% as level 4 (thick purée), levels internationally recognized as safe and suitable for people with dysphagia and suitable for 3D printing. The application of the IDDSI scale eliminated ambiguities in the description of textures, facilitating clear communication between healthcare professionals, caregivers, people with dysphagia, and companies that produce 3D-printed foods, as well as the standardization of 3D food printing processes related to textures. The application of the IDDSI scale eliminated ambiguities in the description of textures, facilitating clear communication between healthcare professionals, caregivers, people with dysphagia, and companies producing 3D-printed food products. This enabled the standardization of 3D food printing processes and the definition of their textures. At the same time, 3D printing proved to be a viable and effective tool for customizing meals that are safe, appropriate, and sensorially appealing. Conclusions: The feasibility of combining the IDDSI scale with 3D printing to develop diets tailored to the needs of people with dysphagia is confirmed. This integrative approach represents an innovation in the field of nutrition for people with swallowing problems, especially in contexts with limited scientific evidence, combining the validation of modified textures with 3D printing technology. There are effective tools for producing safe, suitable and sensorially appealing meals.

## 1. Introduction

Dysphagia is defined as the difficulty or inability to swallow safely and efficiently, and in most cases is related to age-related problems, degenerative processes, or secondary to other pathologies, such as neurological diseases (stroke or Parkinson’s disease), complex tumor surgeries, accidents, etc. [1]). It occurs in up to 93% of people over the age of 64, being particularly prevalent among those who are institutionalized, with an estimated incidence of 80% [2,3]. The consequences of dysphagia include not only malnutrition and dehydration, but also other serious complications such as pulmonary aspiration or aspiration pneumonia, significantly increasing morbidity and mortality. For all these reasons, dysphagia can be considered one of the disorders with the greatest impact on people’s quality of life, especially in the elderly [4].

In response to these clinical challenges, an international consortium was created, the International Dysphagia Diet Standardisation Initiative (IDDSI), led mainly by [5] with the aim of developing a standardized and global system, providing a uniform methodology that is easily applicable to different cultural and clinical contexts in problems related to swallowing. This consortium gave rise to the IDDSI framework, developing and defining in detail the different texture levels and their testing methods to classify solid and liquid foods according to their consistency. The IDDSI scale/system allows for the preparation of foods with modified texture, adapting them to the needs of people with dysphagia or swallowing-related problems and facilitating interprofessional communication (doctors, nutritionists, speech therapists, and kitchen staff), ensuring more coordinated and effective care [6,7,8]. The IDDSI scale covers eight levels, from thin liquids (level 0) to solid foods (level 7), allowing the diet to be adjusted according to the patient’s swallowing abilities [9].

The use of the IDDSI scale in the management of dysphagia has shown significant benefits. Namasivayam et al. conducted a systematic review that showed how modifying food textures based on standards such as IDDSI improved food safety and reduced the risk of aspiration in people with dysphagia [7]. On the other hand, authors such as [10] highlighted that the implementation of this scale made it possible to optimize the sensory experience of modified foods and improve diet adherence and patient satisfaction. Despite the great advantages of this scale, it still faces major challenges related to adequate staff training, the lack of continuing education programs, and the lack of resources to adapt hospital infrastructure [6].

In parallel with advances in the design of standards based on modified food texture, recent research has explored the use of innovative technologies (3D food printing) to improve the quality of life of people with dysphagia. This technique offers the possibility of producing food with different shapes, colors, and flavors, maintaining the nutritional characteristics of the food, and creating textures adapted to each person’s clinical condition [5]. This technological advance mitigates the problems associated with diets traditionally used for people with dysphagia (puréed foods) by reducing dietary monotony, boredom, and lack of pleasure when eating [11]. A pilot study conducted as part of the NutriAlth3D project showed that 3D-printed foods could meet the safety and usability criteria established by the IDDSI and help to enhance the independence and well-being of people with dysphagia and their caregivers [12].

Despite these advances, the scientific literature remains very limited in terms of mixed studies that integrate biological, psychological, and social aspects of dysphagia and the incorporation of emerging technologies [10] From a biopsychosocial perspective, authors such as [13] have highlighted how dysphagia negatively impacts people’s perception of quality of life, generating feelings of social isolation, fear of eating, and low self-esteem [14]. In this context, assessing health-related quality of life (HRQoL) becomes important, as it allows key areas to be identified where interventions can have the greatest impact and support by better personalizing care [15].

Based on this series of research and positions, the present research project aims to analyze the effect of the IDDSI scale integrated with innovative technologies, such as 3D food printing, for people with dysphagia. It seeks to deepen understanding of the use of the IDDSI scale as a standardized framework for the preparation of texture-modified foods, evaluating its effectiveness in conjunction with 3D printing. This objective is part of a broader project that evaluates the potential influence of 3D food printing on the quality of life of people living with dysphagia and their caregivers [11].

## 2. Materials and Methods

This study is part of a broader study, which used a mixed quasi-experimental (qualitative-quantitative) design conducted from October 2021 to November 2023, with the main objective of obtaining a comprehensive view of the impact of 3D food printing on the quality of life of people affected by swallowing disorders. Within the overall project, one of the secondary objectives was to conduct a pilot test with the aim of using the IDDSI scale as a standardized system for measuring modified texture for dysphagia processes and 3D printing. The methodology integrated validation protocols for 3D-printed menus by people with dysphagia and health professionals, using ad hoc and self-developed validated scales, together with international guidelines on texture-modified diets and the use of the International Dysphagia Diet Standardisation Initiative (IDDSI) scale.

All participants had been prescribed and were consuming texture-modified “puréed” diets as part of routine clinical care prior to enrolment in the study. For this reason, and given the pilot and feasibility nature of the project, we adopted a single-arm, within-subject design and did not include a parallel control group receiving conventional puréed meals without 3D printing. The intervention consisted of offering 3D-printed dishes that were nutritionally comparable and adapted to the same IDDSI levels as the participants’ usual diets. Therefore, participants’ previous experience with traditional purée constituted the clinical reference condition against which they subjectively judged the safety, acceptability, and overall satisfaction of the 3D-printed dishes, rather than a formally randomized comparison.

A Natural Machines™ printer was used to print all the food items in the study and pilot test. This printer has space for five separate food capsules and the option of using different nozzle sizes to adapt the printing to the textures to be produced; the intermediate nozzle was used for this study.

Throughout the production process, the dishes were first cooked by expert chefs, then the different parts of the dish were crushed and placed in each of the printer’s capsules. All dishes were printed at room temperature, avoiding extremes of heat or cold, although this temperature was adapted according to the type of dish to be printed. Desserts were normally printed at lower temperatures than starters or main courses. All dishes were prepared in the institutional kitchen of the participating center, following the standard food-safety and hygiene procedures of the facility, which are based on current national regulations and HACCP principles. During cooking, handling, and service, kitchen staff applied the usual time–temperature controls and hygiene measures used for all texture-modified diets in the center. The 3D food-printing step was integrated into this existing circuit as a final plating and shaping stage using cooked, ready-to-eat purée, and the printed dishes were handled and served according to the same food-safety procedures as the rest of the menus for people with dysphagia.

Although the study was not designed to prospectively collect objective clinical outcome measures, all participants continued to be monitored according to the usual clinical protocols of the institution. Throughout the study period, no choking or aspiration events related to meal intake were reported by the nursing or medical staff. All menus were planned and supervised by healthcare professionals (dietitians and nursing staff) to comply with balanced nutritional requirements and to be adapted to older adults with oropharyngeal dysphagia, following the centre’s standards for texture-modified diets.

The project was divided into two phases, with phase 1 consisting of the validation of the menus by experts and Phase 2: testing with participants and the IDDSI classification process.

### 2.1. Phase 1: Expert Validation

The first phase of the project consisted of the design and validation of the 3D-printed dishes (Table 1), with the aim of certifying their safety and suitability for use during the intervention. The validation was carried out by a multidisciplinary team of 26 experts (nutritionists, nurses, speech therapists, 3D engineer, and chefs), using an ad hoc Likert scale (previously validated using the Delphi method) to measure aspects such as swallowing safety (texture, adhesiveness, and risk of aspiration), the sensory properties of the printed foods (taste, smell, temperature, acceptability, and overall satisfaction with the dish), technical compatibility with 3D printing, and viability for use in people with dysphagia.

### 2.2. Phase 2: Testing with Participants and IDDSI Classification Process

In the second phase, the intervention, four independent sessions were held to test all the dishes validated in the previous phase. Forty-eight people participated, according to the established inclusion criteria: people who had been eating orally for at least one month prior to the start of the intervention; labeled with dysphagia type R13.10 (ICD-10), MD93 (ICD-11), or positive dysphagia screening in their medical history, and with dysphagia identified at least three months prior to the start of the intervention, among the most important. The following were excluded from the study: participants with a prognosis of less than six months to live, who did not or could not eat orally at least once a day, and who had a diagnosed cognitive disorder and no caregiver.

For each of the dishes tested in this second phase (Table 1), each participant made an individual and subjective assessment, using an ad hoc scale (the same as in the first phase) together with an open-ended questionnaire of their own design, evaluating the overall degree of satisfaction with the dishes tested. Finally, each of the printed dishes that received a positive assessment from the participants (classified as suitable or very suitable from the point of view of safety for use by people with dysphagia) was analyzed using the IDDSI scale to determine the texture level of each of the foods that composed them.

## 3. Results

### 3.1. Phase 1: Expert Validation

In the first phase, 26 experts (3 nutritionists, 2 nurses, 2 speech therapists, a 3D engineer, and 18 chefs, of whom 16 (62%) were female and 10 (38%) were male), validated 24 different dishes (one starter, one main course, and several desserts) (Table 1), resulting in a total of 142 evaluations of 3D-printed dishes. 85.20% (121 dishes) obtained a positive overall satisfaction rating and were deemed suitable for use by people with dysphagia. In contrast, 14.80% (21 dishes) required some modification to be used in the second phase, in the intervention. No dish was classified as unsuitable.

### 3.2. Phase 2: Testing with Participants and IDDSI Classification Process

In the second phase, 48 participants (Table 2) validated the 24 dishes, obtaining a total of 225 individual ratings.

Ninety-one percent of the ratings (204 dishes) obtained an overall satisfaction rating on a scale from Suitable to Very Suitable for use by people with dysphagia. Nine percent (21 dishes) obtained a rating of Not Suitable or Not Very Suitable for use by people with dysphagia (Table 3). Overall, and with reference to the assessment related to the safety of the dishes for use by people with dysphagia, 98% (221 dishes) were rated as Safe and Very Safe, compared to 2% (4 dishes) that obtained a rating of Not Safe or Not Very Safe (Table 4).

In the results related to the overall evaluation of the dishes analyzed, 9% (N = 21) received a negative evaluation, mainly due to an unpleasant taste of the ingredients used, an inappropriate temperature, or an unattractive visual presentation (Table 3) This evaluation was based on the subjective perspective of the participants, and not all dishes received the same score. In the case of the 2% (N = 4) of dishes that were considered unsafe, the main causes inadequate stickiness or difficulty in swallowing (Table 4).

**Figure 1:**
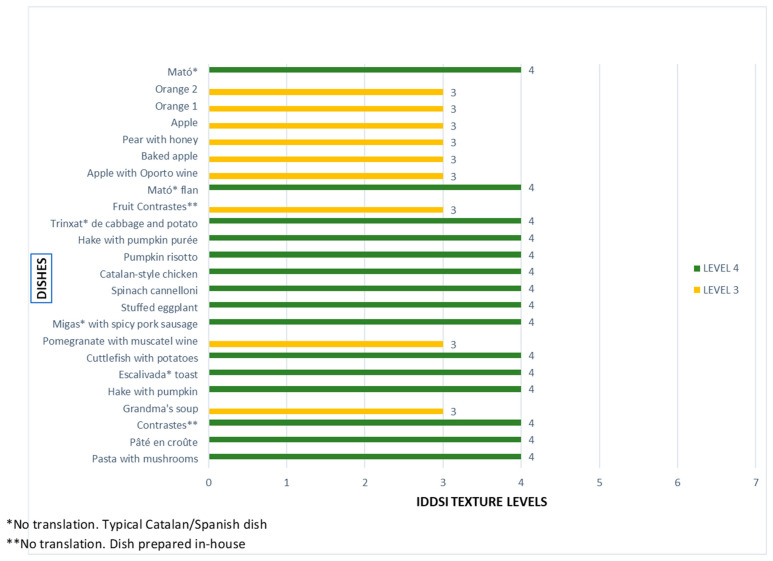
Relationship between texture levels and the dishes analyzed.

In relation to the results obtained with the identification of the texture of 3D-printed dishes using the IDDSI scale, it is important to note that only dishes previously rated as Suitable or Very Suitable for use by people with dysphagia (204 dishes) were analyzed. The data showed that 36% (73 dishes) had a texture level of 3 (Liquidised) and 64% (131 dishes) had a texture level of 4 (Pureed) on the 8-level scale (0 = Thin to 7 = Regular). For more details on the relationship between texture levels and dishes analyzed, see Figure 1.

Once the participants had rated the tested dishes as acceptable and safe, the principal investigator and two collaborators immediately selected a sample of each and carried out the relevant tests to determine the texture level according to the IDDSI scale. All tests to determine the texture level were performed immediately after validation. It was vitally important to do this immediately because the texture and temperature had to be kept as similar as possible to the initial conditions of the food. Each level test always started at the lowest level (level 1) and went up to the level finally reached. For each level, the flow tests indicated by the IDDSI scale itself were always used. For the levels achieved, levels 3 and 4, the flow tests were performed using a syringe (only at level 3), along with drip tests with a fork and tilt tests with a spoon.

According to the results obtained (Figure 1), it can be seen that most preparations are at level 4, indicating that it is possible to transform most traditional main dishes (meat, fish, pasta, rice, and vegetables, such as trinxat, pumpkin risotto, Catalan-style chicken, spinach cannelloni, or pasta with mushrooms) into safe and consistent textures for swallowing. In contrast, a small group of dishes are classified as level 3, mainly desserts and preparations with a high-water content, such as various orange and apple dishes, pear with honey, the “Fruit Contrastes” dessert, pomegranate with muscatel, and sopa de la abuela (grandmother’s soup). These results reveal a clear pattern: savory dishes and main courses are easily adapted to level 4, while some sweet dishes based on fresh fruit and soups tend to maintain a slightly more fluid consistency (level 3), despite remaining compatible with the IDDSI scale recommendations.

## 4. Discussion

The analysis of food texture is a critical element in the development of diets adapted for people with swallowing disorders, such as dysphagia. In this context, standardizing textures using internationally recognized tools is a key strategy for ensuring food safety and nutritional quality [16]. This research used the IDDSI scale as the main tool for validating the textures of dishes designed and 3D printed for people with dysphagia [17].

The IDDSI scale, consisting of eight levels (0–7), provides an objective and reproducible classification for both liquid and solid textures, facilitating its clinical, industrial, and domestic application [18]. In this study, the analysis focused specifically on the levels corresponding to solid foods: level 3 (Liquidised) and level 4 (Pureed), as these levels are widely recognized in scientific literature as suitable for patients with moderate to severe dysphagia, allowing safe swallowing without significant risk of choking or aspiration [19]

Of the 24 dishes analyzed, with a total of 204 validations, 36% were evaluated as suitable at level 3 and 64% at level 4. These findings are consistent with previous studies that highlight the greater acceptability and functionality of dense purée texture (level 4) in clinical contexts and in situations of dysphagia [20]. These results not only exceeded the established safety criteria but also identified the ideal texture for 3D food printing, providing a visually appealing presentation along with a very positive culinary and emotional experience for the end user, which has been shown to have a positive influence on adherence to modified diets [21].

It is important to note that the use of a relatively large sample of dishes (N = 24) together with 204 validations reinforces the internal validity of the analysis. Unlike what has been found in the literature in reference to previous studies [1,21,22,23,24] while previous studies have evaluated a limited number of food products, this work offers a broad overview of the feasibility of using different textures within the IDDSI scale and their applicability with the use of 3D food printing technology. This breadth in the sample and number of validations reinforces the methodological robustness and enables better identification of the optimal level of texture viable for 3D printing and, in turn, obtaining dishes that guarantee the degree of safety for use in people with dysphagia. As Creswell and Clark (2017) point out, adequate sample size is essential in mixed research to ensure descriptive richness and analytical depth, especially when exploring emerging technologies in healthcare contexts [12].

The systematic use of the IDDSI scale in this study helped to overcome historical ambiguities in the description of modified textures, such as “yogurt-like” or “honey-like consistency,” which lack operational precision and are of dubious reliability and uniformity of criteria. This standardization enables effective communication between healthcare professionals, caregivers, and food manufacturers, and even facilitates possible comparisons between other national and international studies and projects, ensuring the correct interpretation and application of modified diets, with the possibility of replicating dishes in a homogeneous manner in the future [7]. Furthermore, the adoption of this scale ensures the replication of results, facilitating continuity in future research in the field of 3D printing of foods with modified texture [5]

It is important to note that all dishes submitted for IDDSI analysis had previously been validated as “suitable” by end users, which reinforces the consistency between participants’ subjective perceptions and the objective characterization of texture. This dual approach, subjective and objective, ensures that the products developed are both technically viable and socially accepted, a key aspect in technologies aimed at human well-being and influential in people’s satisfaction [25]

Although this is a relatively new topic, especially in relation to 3D food printing, the results obtained are consistent with existing evidence on the importance of texture standardization in improving the quality of life of people with dysphagia [1,17,26,27]. Likewise, our data supports the need to continue integrating internationally validated scales such as IDDSI in the development of new food technologies, in order to guarantee their real applicability and positive clinical impact [9,10].

In conclusion, the use of the IDDSI scale in the validation of 3D-printed food textures proves to be an effective tool for standardizing processes, improving food safety, and optimizing the sensory experience of people with dysphagia. The results obtained suggest that level 4 is the most viable from both a technical and clinical point of view. This approach can serve as a basis for future technological developments and care protocols in the field of personalized nutrition for vulnerable groups and those with swallowing problems.

## 5. Conclusions

This study addresses a relevant topic in the field of clinical nutrition: the use of 3D food printing for people with dysphagia, its standardization using the International Dysphagia Diet Standardization Initiative (IDDSI), and patient safety. These three areas have a combined impact on the quality of life of a vulnerable group.

The use of the IDDSI scale is presented as a fundamental element in ensuring reproducibility, objectivity, and safety in the preparation of adapted meals. By integrating this internationally validated tool, subjective descriptions of food textures are overcome, facilitating the use of a uniform nomenclature among healthcare professionals, caregivers, and the food industry. This standard allows for the identification of optimal texture levels viable for 3D printing, facilitates the transfer of best practices, promotes the continuity of similar studies, and encourages future expansion into other areas of healthcare related to modified texture. This pioneering work in the combined application of the IDDSI scale and 3D printing technology in the design of personalized diets contributes to filling a significant gap in the field of therapeutic nutrition. In addition, it provides a solid methodological basis for future research seeking to optimize production processes, expand the variety of textures available, or explore new applications in clinical and domestic settings. It also establishes a solid foundation for future clinical and social applications, promoting a real improvement in the quality of life of people affected by swallowing disorders and their caregivers.

The relevance of the study also lies in its potential to transform the eating experience of people with dysphagia by presenting visually appealing, sensorially stimulating, and clinically tailored food alternatives without compromising safety or functionality.

Furthermore, the methodology used in this research combines scientific rigor, technological innovation, and a person-centered approach. Through three distinct phases (menu design and validation, intervention with active user participation, and texture analysis using the IDDSI scale), the feasibility of using 3D food printing for people with dysphagia was validated.

Finally, our study highlights the importance of integrating standardized scales such as the IDDSI into the development of technological solutions for nutritional care. 3D food printing is emerging as a promising tool for personalizing diets, provided it is accompanied by rigorous and internationally validated technical criteria. These findings are expected to drive future research and intervention policies in the field of nutrition for people with special needs, thereby consolidating a more inclusive, safe, humane model and a useful methodology for healthcare institutions, health professionals, and the food industry. For example, they allow healthcare institutions to reproduce dishes with precisely controlled textures and shapes that are visually similar to the original food, enabling them to offer safer, more appealing, and standardized menus. For institutions, this translates into a reduced risk of aspiration and choking, less variability between shifts and services, better adaptation to protocols and clinical guidelines, and a possible reduction in costs associated with complications, readmissions, and malnutrition. In addition, it improves the center’s image of quality and innovation and can increase the satisfaction of patients, families, and professionals.

From the point of view of healthcare professionals, this allows them to work with a common language and clear objectives: a specific IDDSI level is prescribed, and it is known that the kitchen or external supplier can reproduce it consistently. This provides clinical safety; facilitates coordination between nursing, speech therapy, dietetics, and the kitchen; and allows for better evaluation of the relationship between texture, intake, acceptance, and health or quality of life outcomes. At the same time, it encourages specific training in dysphagia and texture assessment, raising the level of competence of the team and reducing the feeling of uncertainty when dealing with these complex patients.

Finally, for the food and catering industry, it opens up a very defined niche market: developing products and menus standardized by texture levels for institutions and homes. Companies that validate their printed dishes according to IDDSI can offer differentiated, safe solutions that can be easily integrated into the clinical protocols of hospitals, nursing homes, and day centers. This strengthens alliances with the healthcare system, promotes purchasing models based on quality and safety, and positions these companies as key technology partners in the care of people with dysphagia.

## 6. Limitations

This study has several limitations that should be considered when interpreting the findings. First, although the number of dish evaluations was relatively high, the sample of participants with dysphagia was modest (n = 48) and was determined by the design and saturation criteria of the broader mixed-methods project. Consequently, the identification of the texture of 3D-printed foods using the IDDSI scale should be considered a pilot, descriptive analysis, with limited statistical power to perform inferential comparisons or subgroup analyses. Future studies should include larger samples to allow more robust quantitative validation of the proposed dishes and texture classifications.

Second, the participants were recruited from a specific healthcare and cultural context and evaluated a finite set of menus developed within this project. This may limit the generalization of the results to other populations, institutions, health systems, and culinary traditions, as well as to other IDDSI levels beyond 3 and 4. Therefore, the extrapolation of these findings to different settings or types of preparations should be made with caution.

Third, both the expert assessments in Phase 1 and the end-user evaluations in Phase 2 were based on subjective perceptions of safety, sensory characteristics, and global satisfaction, collected through ad hoc scales and self-reports. Although these instruments were developed and refined through expert consensus, the reliance on subjective ratings may introduce evaluation bias (e.g., social desirability or expectation bias), and no objective instrumental measurements of texture (e.g., rheological or flow properties) were obtained. Combining subjective evaluations with objective measurements would improve the robustness of future studies.

In addition, the dimension labelled “safety” in our ad hoc rating scales referred specifically to swallowing safety (texture, adhesiveness, and perceived risk of choking or aspiration) and did not include microbiological food safety. No specific microbiological analyses of the 3D-printed dishes were performed as part of this pilot study, and indicators such as microbial counts of finished products or environmental samples were not prospectively collected as research variables. Although food preparation and service followed the institution’s routine food-safety and hygiene procedures, the absence of objective microbiological data represents an important limitation when translating these findings into clinical practice. Future studies should explicitly incorporate microbiological endpoints and systematic monitoring of critical time–temperature points during and after printing to fully validate the safety of 3D-printed, texture-modified diets.

Finally, the study did not include long-term follow-up of participants. The dishes were evaluated in single sessions, without assessing sustained adherence to 3D-printed menus, long-term effects on nutritional status, clinical outcomes (e.g., aspiration events, hospitalizations), or health-related quality of life. Future research should incorporate longitudinal designs to explore whether the integration of IDDSI-standardized 3D-printed foods into everyday diets produces stable benefits over time for people living with dysphagia and their caregivers.

## Figures and Tables

**Table 1:** Types of plates tested.

STARTERS	MAIN COURSE	DESSERT
Pasta with mushrooms	Pâté en croûte	Apple
Grandma’s soup	Hake with pumpkin purée	Mató * flan
Contrastes **	Cuttlefish with potatoes	Orange 1
Escalivada * toast	Migas * with spicy pork sausage	Orange 2
Stuffed eggplant	Catalan-style chicken	Mató *
Pumpkin risotto	Spinach cannelloni	Pomegranate with muscatel wine
Trinxat * with cabbage and potato	Hake with pumpkin	Pear with honey
		Fruit Contrastes **
		Baked apple
		Apple with Oporto wine

* No translation. Typical Catalan/Spanish dish; ** No translation. Dish prepared in-house.

**Table 2:** Demographic data of phase 2 participants.

Female				Male			
<18	18–40	41–64	≥65	<18	18–40	41–64	≥65
0 (0%)	5 (10%)	20 (42%)	13 (27%)	0 (0%)	0 (0%)	1 (2%)	9 (19%)
38 (79%)				10 (21%)			
48 (100%)							

**Table 3:** List of results in the overall assessment of the dishes analyzed.

	N	%
Suitable/Very suitable	204	91.0%
Not at all/Not very suitable	21	9.0%
Total	225	100.0%

**Table 4:** List of safety results for the dishes analyzed.

	N	%
Safe/Very Safe	221	98.0%
Nothing/Not very safe	4	2.0%
Total	225	100.0%

## Data Availability

All data presented in the article are freely available. If further information is required, please contact the corresponding authors.

## Abbreviations

The following abbreviations are used in this manuscript:

IDDSI	The International Dysphagia Diet Standardisation Initiative
HRQoL	Health-Related Quality of Life
ICD11	International Classification of Diseases 11th Revision
HACCP	Hazard Analysis and Critical Control Points
